# A ubiquitin-related gene signature for predicting prognosis and constructing molecular subtypes in osteosarcoma

**DOI:** 10.3389/fphar.2022.904448

**Published:** 2022-08-17

**Authors:** Nan Wei, Gong Chao-yang, Zhou Wen-ming, Lei Ze-yuan, Shi Yong-qiang, Zhang Shun-bai, Zhang Kai, Ma Yan-chao, Zhang Hai-hong

**Affiliations:** ^1^ Orthopaedics Key Laboratory of Gansu Province, Lanzhou, China; ^2^ Lanzhou University Second Hospital, Lanzhou, China

**Keywords:** osteosarcoma, ubiquitin, ubiquitination, immune, molecular subtypes

## Abstract

**Background:** Ubiquitination is medicated by three classes of enzymes and has been proven to involve in multiple cancer biological processes. Moreover, dysregulation of ubiquitination has received a growing body of attention in osteosarcoma (OS) tumorigenesis and treatment. Therefore, our study aimed to identify a ubiquitin-related gene signature for predicting prognosis and immune landscape and constructing OS molecular subtypes.

**Methods:** Therapeutically Applicable Research to Generate Effective Treatments (TARGET) was regarded as the training set through univariate Cox regression, Lasso Cox regression, and multivariate Cox regression. The GSE21257 and GSE39055 served as the validation set to verify the predictive value of the signature. CIBERSORT was performed to show immune infiltration and the immune microenvironment. The NMF algorithm was used to construct OS molecular subtypes.

**Results:** In this study, we developed a ubiquitin-related gene signature including seven genes (UBE2L3, CORO6, DCAF8, DNAI1, FBXL5, UHRF2, and WDR53), and the gene signature had a good performance in predicting prognosis for OS patients (AUC values at 1/3/5 years were 0.957, 0.890, and 0.919). Multivariate Cox regression indicated that the risk score model and prognosis stage were also independent prognostic prediction factors. Moreover, analyses of immune cells and immune-related functions showed a significant difference in different risk score groups and the three clusters. The drug sensitivity suggested that IC50 of proteasome inhibitor (MG-132) showed a notable significance between the risk score groups (*p* < 0.05). Through the NMF algorithm, we obtained the three clusters, and cluster 3 showed better survival outcomes. The expression of ubiquitin-related genes (CORO6, UBE2L3, FBXL5, DNAI1, and DCAF8) showed an obvious significance in normal and osteosarcoma tissues.

**Conclusion:** We developed a novel ubiquitin-related gene signature which showed better predictive prognostic ability for OS and provided additional information on chemotherapy and immunotherapy. The OS molecular subtypes would also give a useful guide for individualized therapy.

## Introduction

Osteosarcoma (OS) serves as the most common primary solid malignancy of bone in children and adolescents. Its characteristic is higher malignant potential and occurrence of metastases (Botter et al., 2014; Kansara et al., 2014). At present, the combination of surgery and adjuvant chemotherapy is considered a potential therapeutic approach and has also improved the overall survival of OS (Harrison et al., 2018). However, gradually increasing metastatic and relapsed OS has been a new threat to the prognosis of OS (Meazza and Scanagatta, 2016). The study reported that the long-term survival rate of metastatic OS was <25% (Anwar et al., 2020). Hence, it is crucial and necessary to develop more effective novel agents and novel therapeutic targets.

Post-translational modification (PTM) is an important step to regulate various kinds of cellular processes, including phosphorylation, ubiquitination, acetylation, and other modifications (Chen et al., 2020). Amongst these PTMs, dysregulation of ubiquitination has been involved in multiple cancers (Nakayama and Nakayama, 2006). Generally, ubiquitination is medicated by three classes of enzymes, such as ubiquitin-activating enzymes (E1), ubiquitin-conjugating enzymes (E2), and ubiquitin ligases (E3) (Hershko and Ciechanover, 1998). Meanwhile, dysregulation of ubiquitination has received a growing body of attention in OS tumorigenesis and treatment. For example, Zhou et al. (2020) reported that the E3 ubiquitin ligase tripartite motif 7 (TRIM7) regulates metastasis and chemoresistance in OS through ubiquitination of breast cancer metastasis suppressor 1 (BRMS1). In addition, Zhang et al. (2017) verified that ubiquitin-conjugating enzyme E2 variant 1A (UEV1A) played a suppressive role in OS differentiation by promoting Smurf1-mediated Smad1 ubiquitination. Taken together, increasing evidence has shown that ubiquitination is closely associated with OS initiation and progression. However, the studies on ubiquitin-related gene signatures and ubiquitin-related molecular subtypes for OS still remain limited.

In this study, we constructed a ubiquitin-related gene signature including seven ubiquitin-related genes through the Therapeutically Applicable Research to Generate Effective Treatments (TARGET) and Gene Expression Omnibus (GEO) database. Meanwhile, we confirmed that the risk score model has good performance in predicting the overall survival and was an independent prognostic factor. Based on the risk score, we evaluated the immune infiltration and chemotherapy drug sensitivity between high- and low-risk groups. Importantly, we also developed novel molecular subtypes for OS by prognostically significant ubiquitin-related genes.

## Materials and methods

### Data processing

**FIGURE 1:**
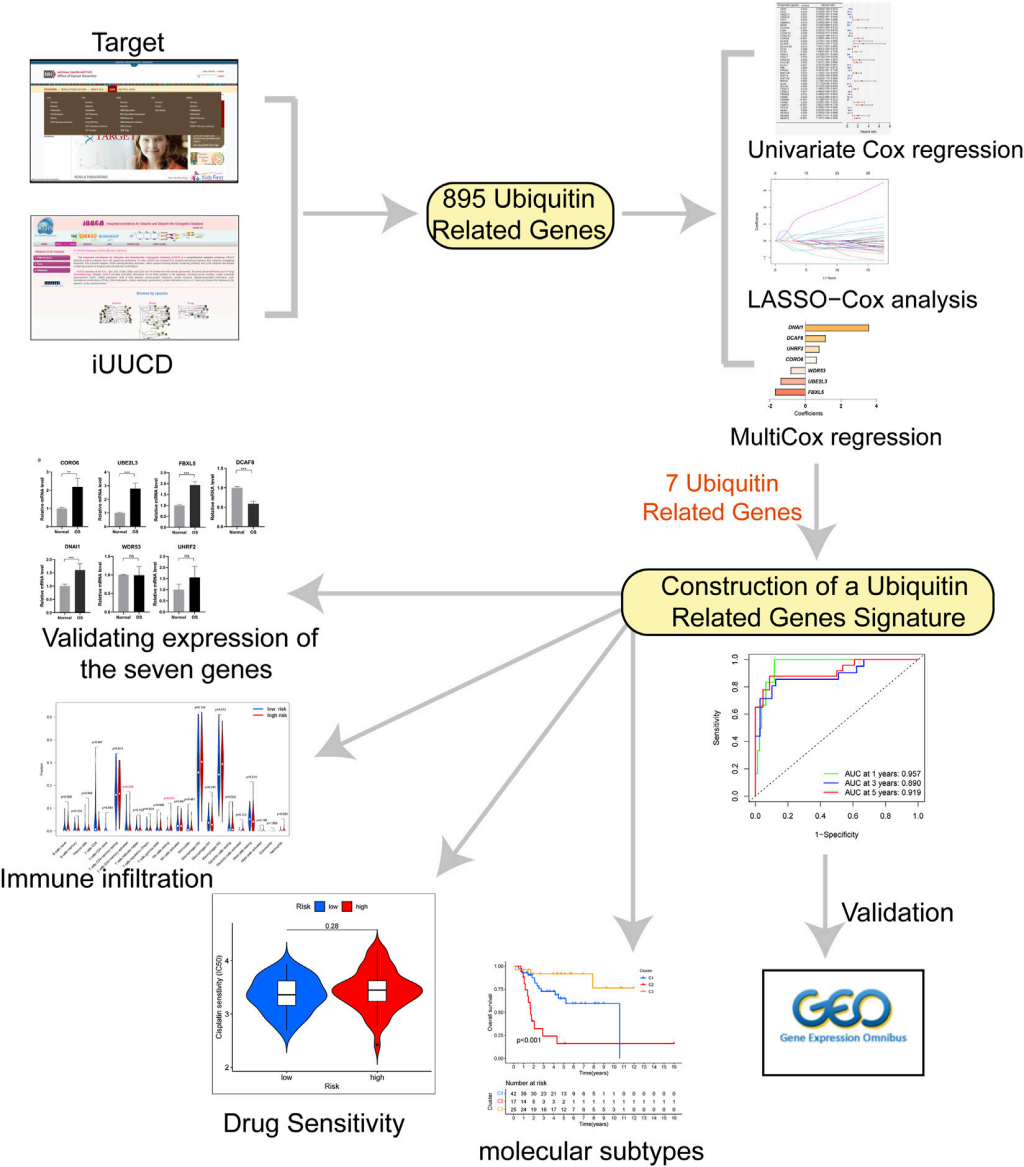
The flow chart for this study.

The study included three datasets (Table 1). RNA sequence data and clinical follow-up information of the training set were downloaded from public databases: the Therapeutically Applicable Research to Generate Effective Treatments (TARGET; https://ocg.cancer.gov/programs/target). Then, FPKM values were transformed into TPM values through the R package “dplyr” (Mangiola et al., 2021). Gene expression matrix and clinical follow-up information of validation set (GSE21257 and GSE39055) were downloaded from GEO (https://www.ncbi.nlm.nih.gov/geo/). The workflow of the study is shown in Figure 1. The code of analysis was showed in Supplementary Data Sheet 1.

**TABLE 1:** Clinical characteristics for all patients.

Characteristics	Groups	Training set (TARGET) (n, %)	GSE21257 (n, %)	GSE39055 (n, %)
Age	<16 years	48 (57.1)	23 (43.4)	29 (78.4)
	≥16 years	36 (42.9)	30 (56.6)	8 (21.6)
Gender	Female	36 (42.9)	19 (35.8)	17 (45.9)
	Male	48 (57.1)	34 (64.2)	20 (54.1)
Metastatic	Metastatic	21 (25.0)	34 (64.2)	18 (48.6)
	Non-metastatic	63 (75.0)	19 (35.8)	19 (51.4)
Status	Alive	55 (65.5)	30 (56.6)	27 (73.0)
	Dead	29 (34.5)	23 (43.4)	10 (27.0)

### Construction and evaluation of a ubiquitin-related gene signature

We acquired 911 ubiquitin-related genes from the integrated annotations for Ubiquitin and Ubiquitin-like Conjugation Database (iUUCD, http://iuucd.biocuckoo.org/browse.php). Finally, we obtained the expression matrix of 895 ubiquitin-related genes from the TARGET dataset. Then, univariate Cox regression and Lasso Cox regression were used to select prognosis-related ubiquitin-related genes by using R packages “survival” (Lorent et al., 2014) and “glmnet” (Engebretsen and Bohlin, 2019). Multivariate Cox regression was performed to obtain the risk score and risk classification of each OS case. The algorithm formula was as follows: *Risk score = (Exp of gene1 x Coefficient of gene 1)+(Exp of gene 2 x Coefficient of gene 2)+…+(Exp of gene N x Coefficient of gene N).*

The receiver-operating characteristic (ROC) curves were used to evaluate the diagnostic efficacy of the risk score model through the “timeROC” (Blanche et al., 2013) software package. Based on a ubiquitin-related gene signature, the principal component analysis (PCA) was applied to show distribution patterns of OS case by using the “ggplot2” software package (Gómez-Rubio, 2017). The Kaplan–Meier curve analysis was performed to evaluate overall survival in the high- and low-risk group by using R package “survival” package. Moreover, multivariate Cox regression was applied to evaluate the independent prognostic value through R package “forestplot”.

### Construction nomogram

In order to predict overall survival probability at 1, 3, and 5 years for osteosarcoma patients in the Target cohort, we constructed a nomogram to evaluate the probable overall survival of the OS patients through the R package “rms” (Bandos et al., 2009).

### Immune cell infiltration and immune microenvironment

The CIBERSORT (Newman et al., 2019) was applied to assess the difference in the expression matrix of immune cells in the high- and low-risk group. Meanwhile, we compared the immune microenvironment (stromal score, immune score, and ESTIMATE score) by using the R package “estimate” (Yoshihara et al., 2013) in Cluster 1, Cluster 2, and Cluster 3.

### Gene set enrichment analysis, Gene Ontology, and Kyoto Encyclopedia of Genes and Genomes

GSEA was performed to evaluate the significant biological functions between high- and low-risk groups through the R package “clusterProfiler” (Yu et al., 2012), and the significant enrichment was selected with | NES | > 1 and *p* value < 0.05. In addition, the annotated gene sets (“c5. go.v7.4. symbols.gmt” and “c2. cp.kegg.v7.4. symbols.gmt”) served as the reference set. The prognosis-related ubiquitin-related genes were used to conduct GO and KEGG enrichment analyses by using the R package “clusterProfiler.” The significant criterion was *p* value < 0.05.

### Prediction of drug sensitivity and nonnegative matrix factorization clustering

In order to further explore the clinical reliability of the risk score model, we predicted the half-maximal inhibitory concentration (IC50) of chemotherapy drugs through R package “pRRophetic” (Geeleher et al., 2014). Moreover, the “NMF” method was applied to identify OS subtypes through the R package “NMF” (Lee and Seung, 1999).

### Quantitative real-time PCR

A total of six osteosarcoma and six normal tissues were obtained from 12 patients at the Department of Orthopaedics, Lanzhou University Second Hospital in June 2022, and this research was supported by the Independent Ethics Committee (IEC). Total RNA from the tissues was extracted by the TRIzol (AG21101, Hunan, China). Reverse transcription was carried out by using the Evo M-MLV RT Kit (AG11705, Hunan, China). cDNA amplification was carried out by using SYBR® Green Premix Pro Taq HS qPCR Kit (AG11718, Hunan, China). GAPDH was used as an internal control. All gene primers were listed in Supplementary Table 2.

### Statistical analysis

All statistical analyses and figure visualization were used by R software (version 4.0.2) in this study. Perl (version 5.8.3) was applied to combine gene expression data with clinical information. The *p* value < 0.05 was considered statistical significance.

## Results

### Construction of a ubiquitin-related gene signature in osteosarcoma

**FIGURE 2:**
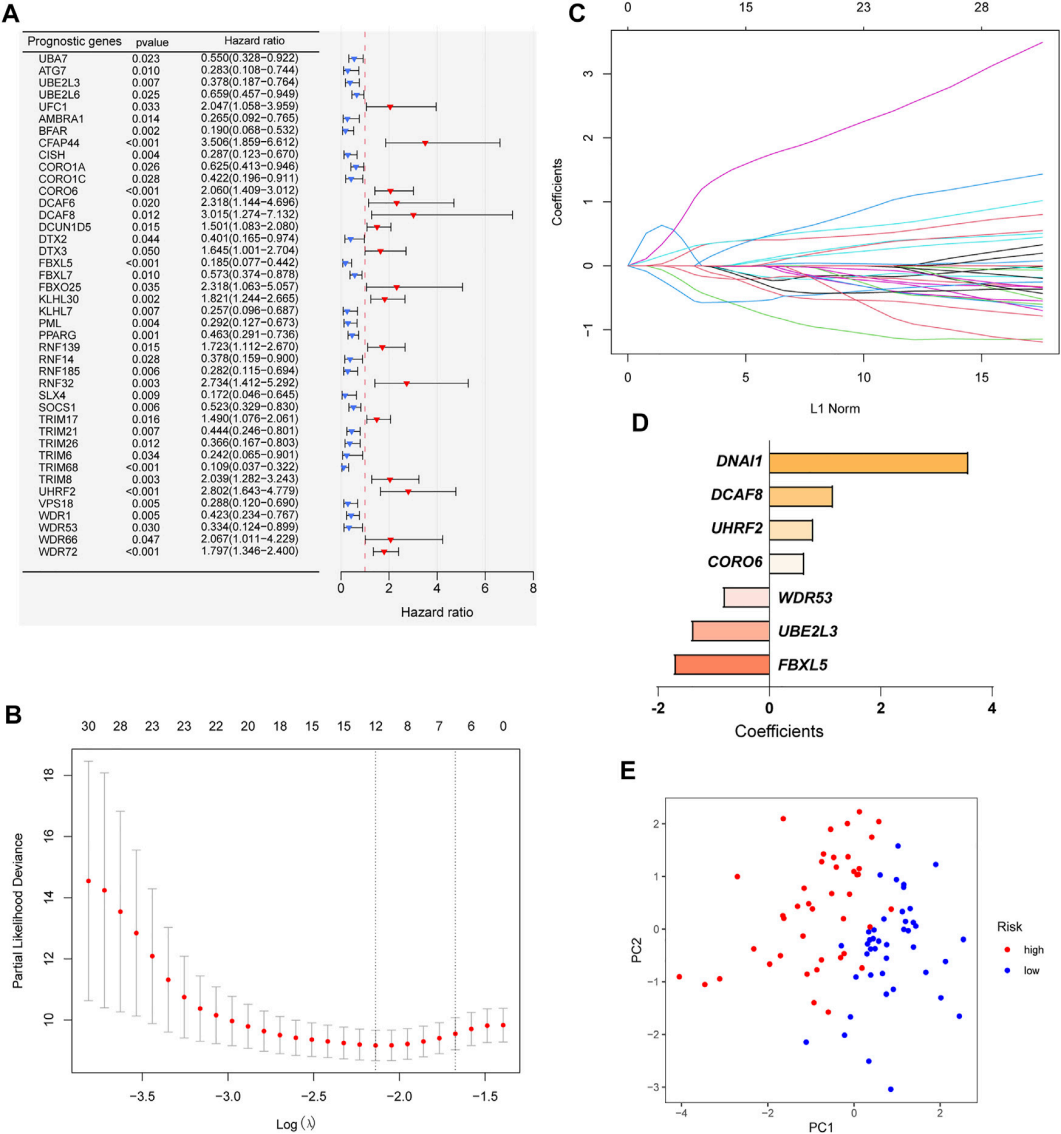
Construction of a ubiquitin-related gene signature. **(A)** Identifying 42 ubiquitin-related genes involved with the overall survival through Univariate Cox regression analysis. **(B–C)** Twelve ubiquitin-related genes were screened through Lasso Cox regression analysis. **(D)** Coefficients of the seven ubiquitin-related genes through multivariate Cox analyses. **(E)** Principal component analysis (PCA) based on the signature.

In order to construct a ubiquitin-related gene signature, we firstly identified 42 ubiquitin-related genes that were significantly associated with the overall survival of OS patients through univariate Cox regression (Figure 2A). Meanwhile, we also found that the expression of 26 ubiquitin-related genes was positively correlated with overall survival, and others were negative. Then, lasso Cox regression was applied to further screen candidate genes of ubiquitin-related gene signature. The results showed that 12 ubiquitin-related genes were regarded as good candidates (Figures 2B,C). Eventually, we screened the seven ubiquitin-related genes (UBE2L3, CORO6, DCAF8, DNAI1, FBXL5, UHRF2, and WDR53) to construct prognostic signature through multivariate Cox regression. The coefficient of each gene was shown in Figure 2D. Based on the results of PCA, we can find that all OS cases were well divided into different distribution patterns (Figure 2E).

### Evaluation and validation of a ubiquitin-related gene signature

**FIGURE 3:**
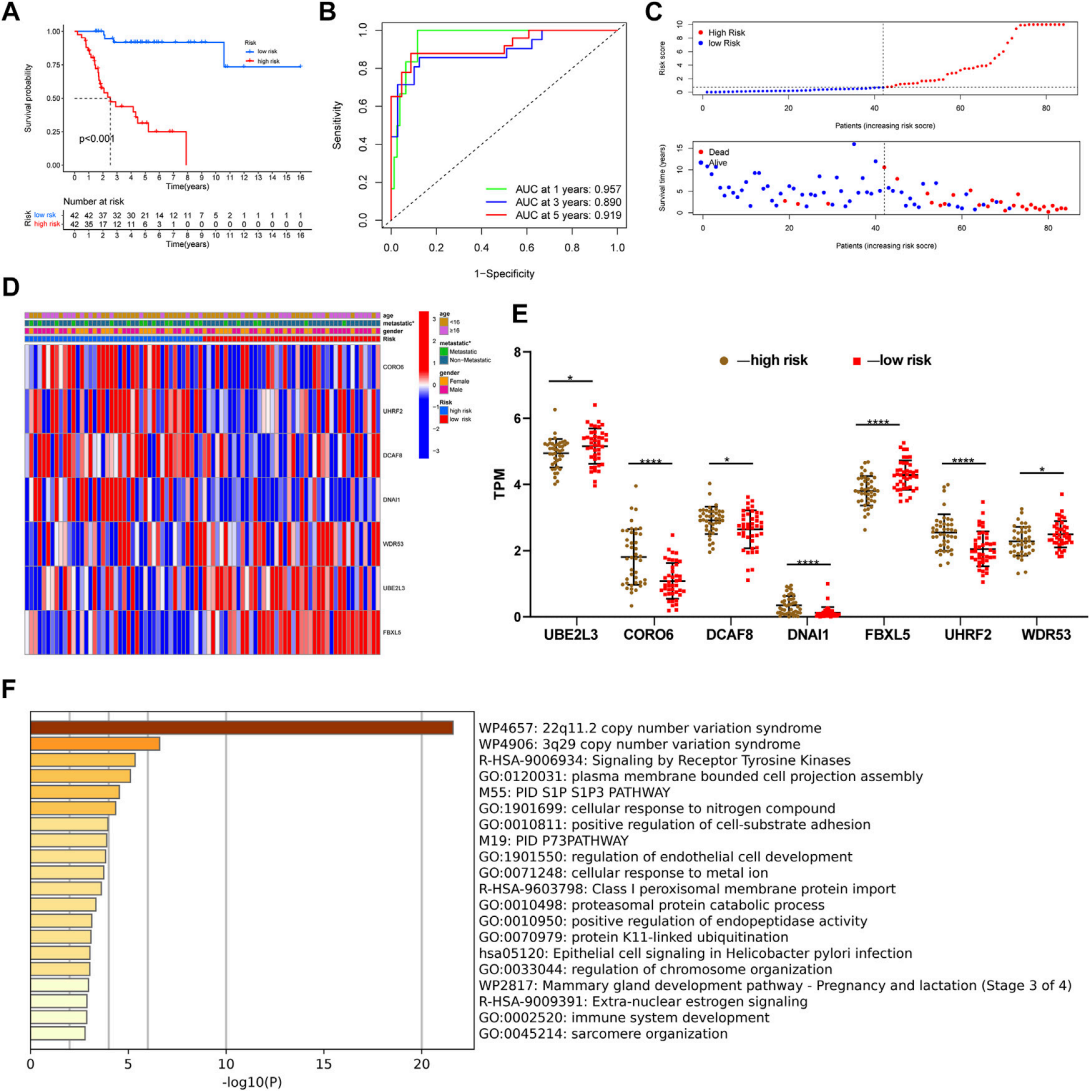
Evaluating a ubiquitin-related gene signature. **(A)** The Kaplan–Meier survival curve in the Target set. **(B)** The ROC curves in the Target set. **(C)** Risk score distribution and survival status of OS patients in the Target set. **(D)** Heatmap of seven ubiquitin-related genes with combining clinical information in the Target set. **(E)** Differential expression of these seven genes between high and low-risk groups. **(F)** Functional annotation of co-expression genes through the metascape. *p < 0.05, **p < 0.01*,* ***p < 0.001, ****p < 0.0001.

**FIGURE 4:**
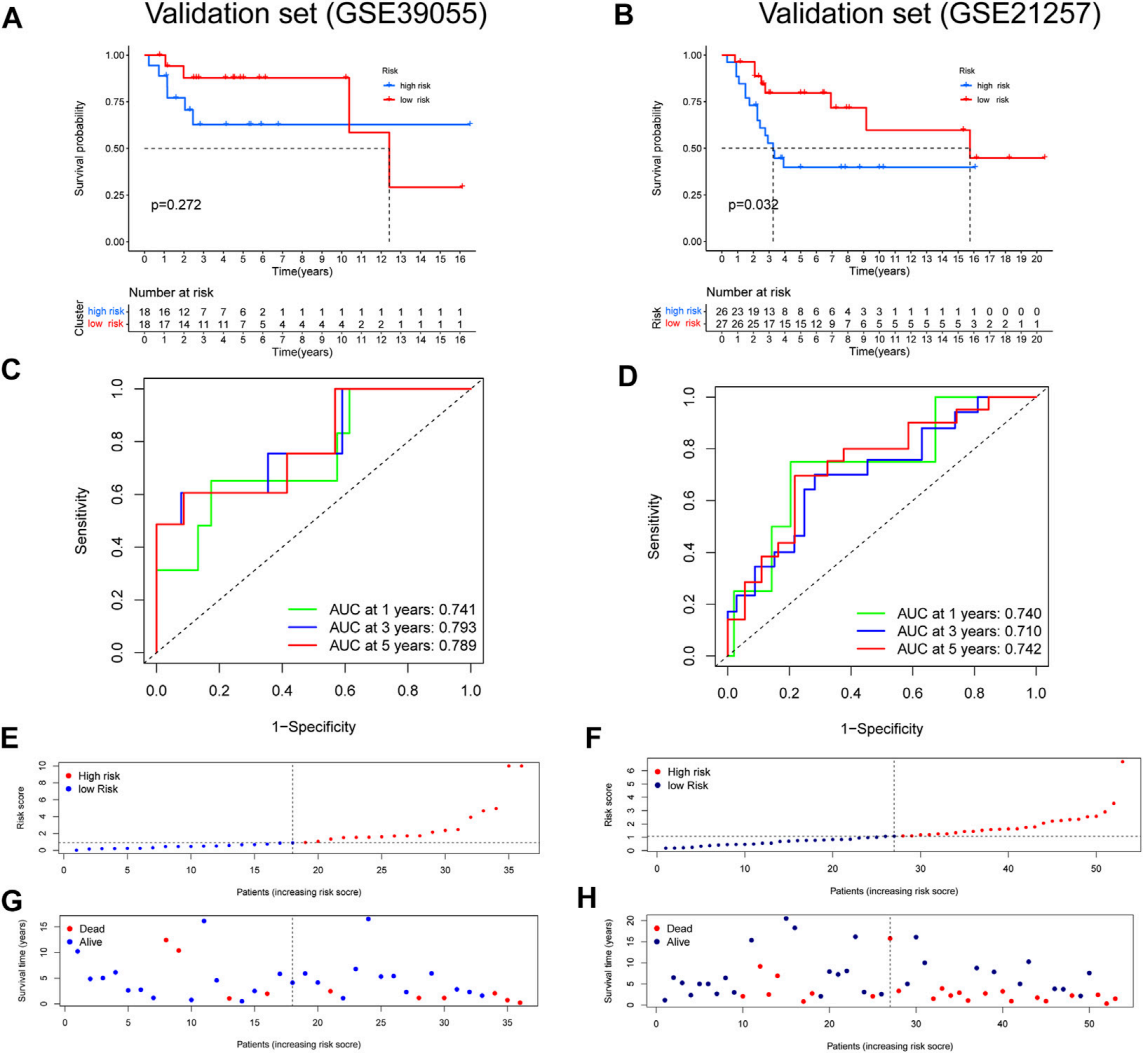
Validation of a ubiquitin-related gene signature in two independent cohorts. **(A,B)** The Kaplan–Meier survival curve in two validation sets. **(C,D)** The ROC curves in the validation sets. **(E-H)** Risk score distribution and survival status of OS patients in the validation sets.

After constructing a ubiquitin-related gene signature, all samples were divided into high-risk and low-risk groups. The Figure 3E showed the differential expression of these seven genes between high and low-risk groups. The Kaplan–Meier survival curve suggested that there was a significantly difference in overall survival between the high- and low-risk groups in the Target set (Figure 3A) and validation (GSE21257) set (Figure 4B). Further, the ROC curves presented good prediction efficiency for OS overall survival in the Target set and validation set (Figure 3B and Figures 4C,D). And in the Target set, the respective AUC values at 1/3/5 years were 0.957, 0.890, and 0.919. Moreover, risk score distribution and survival status of OS patients were shown in (Figure 3C and Figures 4E–H), and we constructed the heatmap of seven ubiquitin-related genes by combining clinical information and risk score groups in the Target set (Figure 3D). To further explore the function of seven ubiquitin-related genes, we performed a co-expression analysis of these seven genes in the data matrix containing all genes and obtained the 226 co-expression genes with seven ubiquitin-related genes in the Supplementary Table 3 (R > 0.5, *p* < 0.001). Functional annotation of co-expression genes showed in Figure 3F through the Metascape. Taken together, the above results proved that the signature had a good performance in predicting prognostic efficiency.

### Assessment of the prognostic value of the risk score model and establishment of the nomogram

**FIGURE 5:**
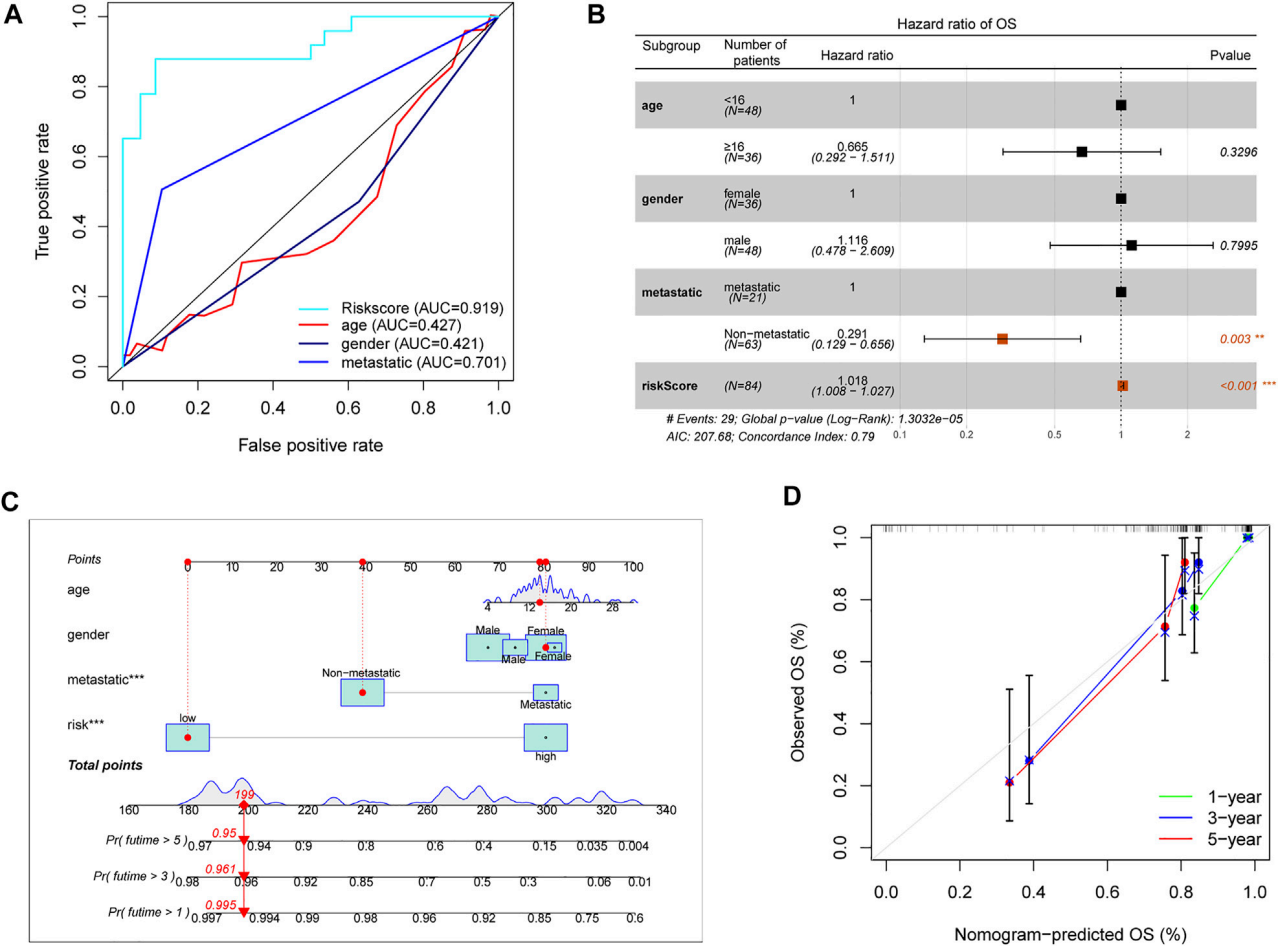
Assessment prognostic value of risk score model and the nomogram. **(A)** The prediction ability of the risk score model and other clinicopathological features. **(B)** Analyses of prognostic prediction factors through multivariate Cox analyses. **(C)** Nomogram for predicting 1/3/5-years survival rates of OS patients. **(D)** 1/3/5-year calibration curve of the nomogram.

As above described, the risk score model had an advantage in prediction efficiency. Then, we compared the risk score model with other clinicopathological features. The ROC curves showed that the prediction ability of the risk score model was better than other clinicopathological features, including age (AUC = 0.427), gender (AUC = 0.421), and prognosis stage (AUC = 0.701) (Figure 5A). Moreover, multivariate Cox regression indicated that the risk score model and prognosis stage were also independent prognostic prediction factors (Figure 5B). Additionally, a nomogram with a risk score model and other clinicopathological features was constructed to better predict the survival rate of OS patients (Figure 5C). Based on the nomogram, we can obtain the survival rate of 1/3/5 years by calculating the total points of every patient. Furthermore, the 1/3/5 years calibration curve was shown in Figure 5D. Overall, data showed that the risk score model had a better advantage in prognostic prediction than other clinicopathological factors.

### Correlation between immune and risk score model

**FIGURE 6:**
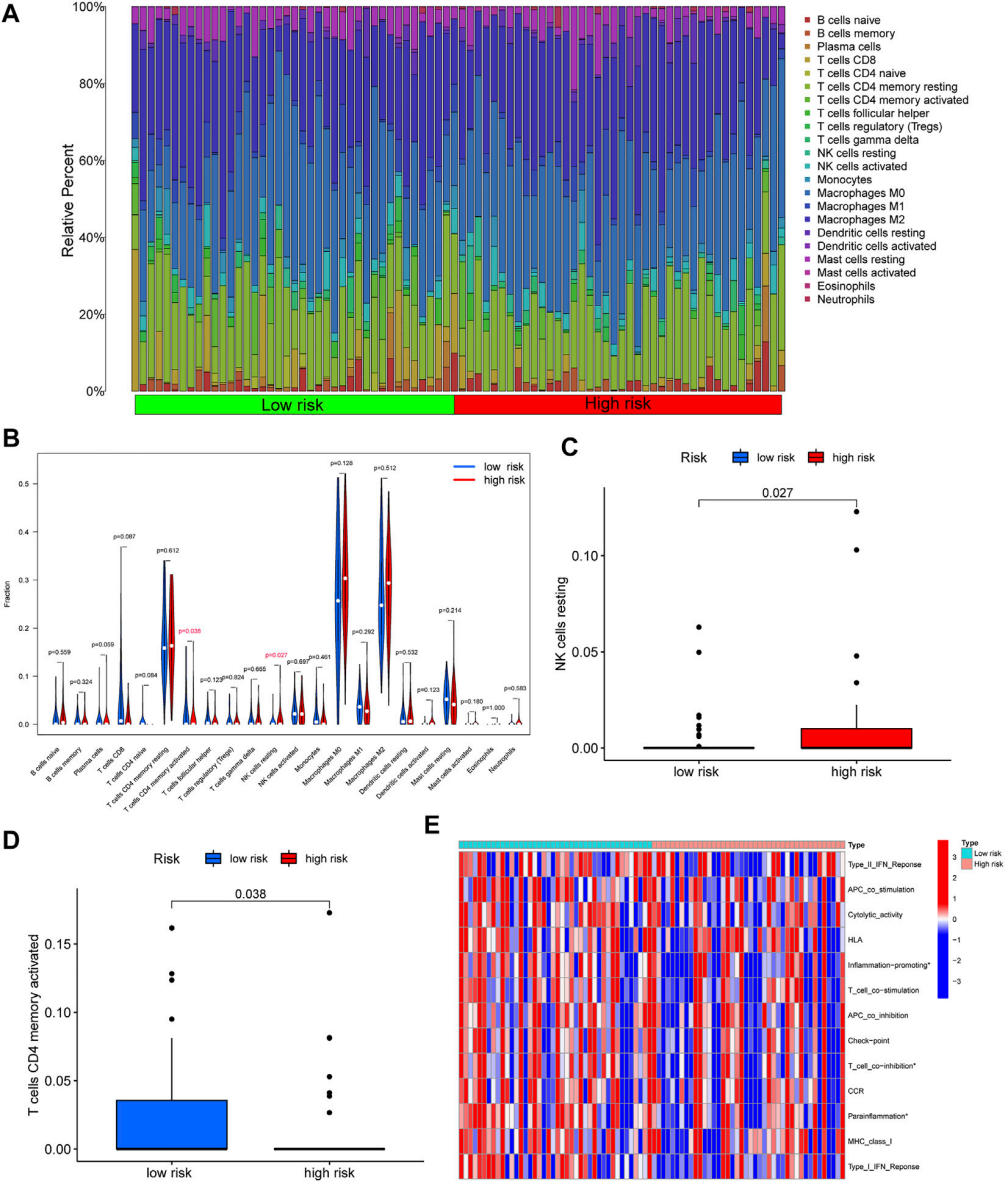
Correlation between immune and risk score model. **(A)** The 22 immune cells landscape between high- and low-risk groups. **(B)** Comparison with the percentage of immune cells in the high- and low-risk groups. **(C–D)** A significant percentage of T cells CD4 memory activated and NK cells resting. **(E)** The significant immune-related functions in the different risk score groups (^∗^ *p* < 0.05).

In order to further explore the correlation between the immune and risk score model, we carried out the analyses of immune cells and immune-related functions in the matrix of seven genes after screening and the reference dataset of CIBERSORT. As shown in Figure 6A, we described the 22 immune cells landscape between high- and low-risk groups. The results showed that the percentage of T cells CD4 memory resting, Macrophages M0, and Macrophages M2 were higher than others in OS samples. Furthermore, the fraction of T cells CD4 memory activated (*p* = 0.035) and NK cells resting (*p* = 0.027) had a significant difference in the high- and low-risk groups (Figures 6B–D). Moreover, according to the result of immune-related functions, we found that inflammation-promoting, T cell co-inhibition, and parainflammation were significantly different in the risk score groups.

### GSEA, GO, and KEGG

GSEA was applied to explore significant biological functions and pathways in the risk score groups.

**FIGURE 7:**
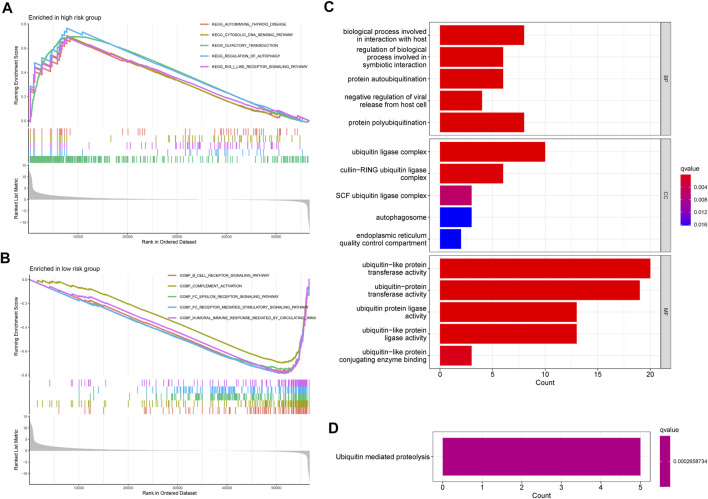
GSEA, GO, and KEGG. **(A)** The GSEA showed significant enrichment in the low-risk groups. **(B)** The GSEA showed significant enrichment in the low-risk groups. **(C)** The significant GO terms are based on the prognosis-related ubiquitin-related genes. **(D)** The significant KEGG pathways are based on the prognosis-related ubiquitin-related genes.

As shown in Figure 7A, the top five enrichment of signaling pathways, such as cytosolic DNA sensing pathway, regulation of autophagy, RIG I like receptor signaling pathway were significantly enriched in the high-risk groups. Furthermore, according to the result of enrichment analysis, significant biological functions including B cell receptor signaling pathway, complement activation, FC epsilon receptor signaling pathway, FC epsilon receptor-mediated stimulatory signaling pathway, humoral immune response mediated circulating immune signaling pathway were significantly enriched in the low-risk groups (Figure 7B). Taken together, we can find that most of the GSEA were related to immune between high- and low-risk groups. Moreover, GO and KEGG were used to explore the biological functions and pathways of the prognosis-related ubiquitin-related genes. The top five enrichment of GO and KEGG were shown in Figures 7C,D.

### Chemotherapy drug sensitivity

**FIGURE 8:**
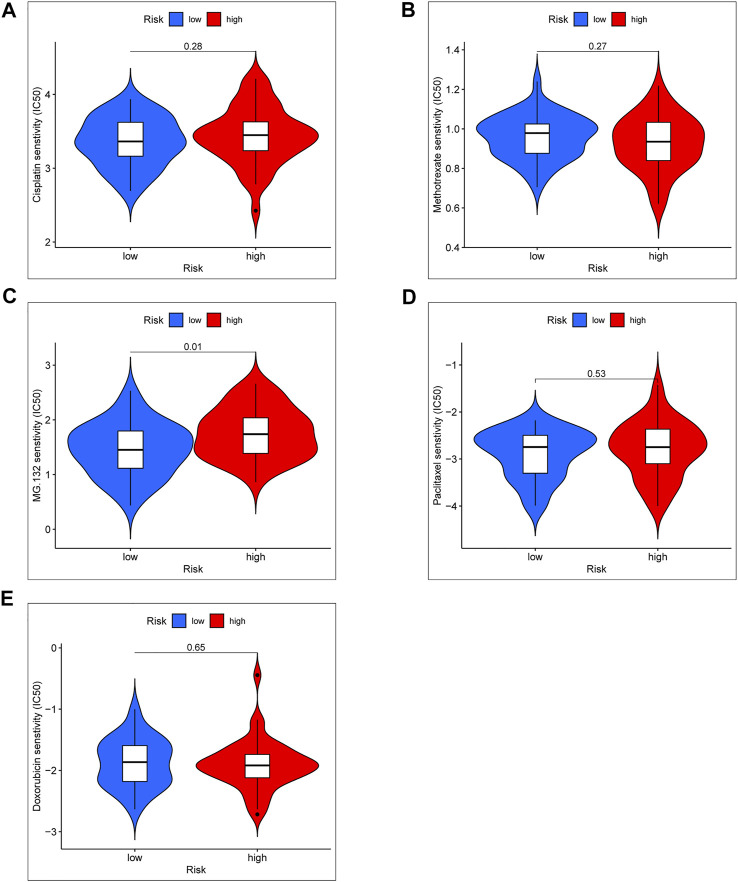
Chemotherapy drug sensitivity between the high- and low-risk group. **(A)** IC50 of Cisplatin. **(B)** IC50 of Methotrexate. **(C)** IC50 of MG-132. **(D)** IC50 of Paclitaxel. **(E)** IC50 of Doxorubicin.

Standard chemotherapy for OS patients included doxorubicin, cisplatin, methotrexate, and paclitaxel (Patel et al., 1996). In order to further explore the clinical significance of the risk score model, we especially analyzed the drug sensitivity of the above chemotherapy drug and a proteasome inhibitor (MG-132) between high- and low-risk groups (Figures 8A–E). According to the results of chemotherapy drug sensitivity, half-maximal inhibitory concentration (IC50) of doxorubicin, cisplatin, methotrexate, and paclitaxel were not significant in different risk groups. Interestingly, IC50 of MG-132 was notable significance between high- and low-risk groups. Based on the result, we proposed a hypothesis that MG-132 may be a novel candidate for OS chemotherapy.

### Different immune microenvironment and immune-related functions in the osteosarcoma subtypes

**FIGURE 9:**
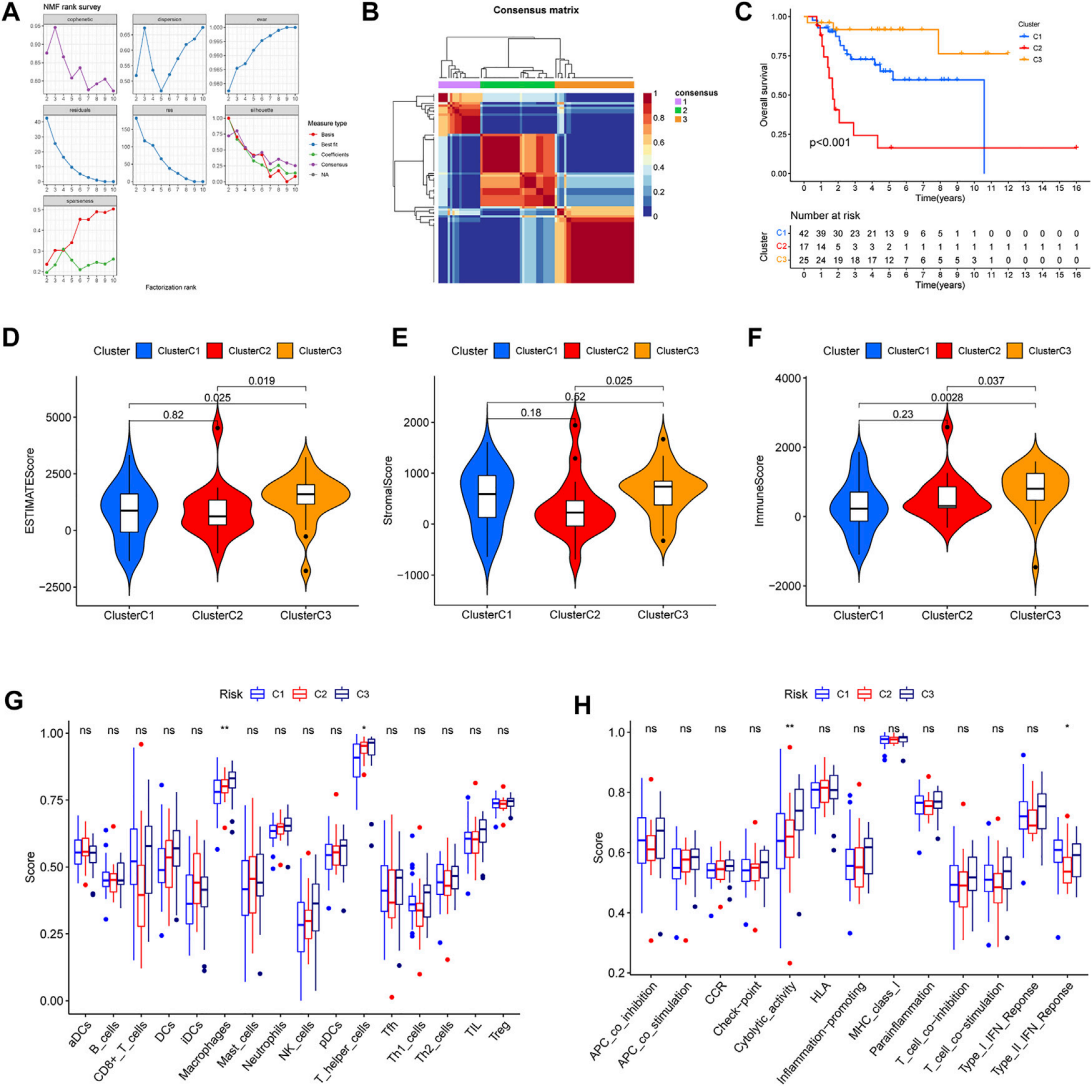
Different immune microenvironment and immune-related functions in the osteosarcoma subtypes. **(A)** The value of the nonnegative matrix factorization (NMF). **(B)** The NMF clustering-based value of best cophenetic. **(C)** Kaplan–Meier curves of OS in the three clusters. **(D–F)** The ESTIMATE score, stromal score, and immune score of OS subtypes. **(G)** The percentage of immune cells in the three clusters. **(H)** The significant immune-related functions in the three clusters.

Furthermore, in order further to explore ubiquitin-related genes, we developed novel OS subtypes. First, 42 prognosis-related ubiquitin-related genes were selected to cluster. Based on NMF algorithm, when the cophenetic was 3, we eventually obtained molecular subtypes of three cluster (Figure 9A). In addition, the cluster map was shown in Figure 9B. Meanwhile, we also made a comparison of prognosis and immune with three clusters. The Kaplan–Meier survival curve showed that cluster 2 had the worst survival outcomes than cluster 1 and cluster 3 (Figure 9C). In addition, we also analyzed the difference in the immune microenvironment (stromal score, immune score, ESTIMATE score), and the results indicated that the stromal score, immune score, ESTIMATE score of cluster 3 was higher than cluster 2 (Figures 9D–F). Additionally, differences in immune cell infiltration and immune-related functions were shown in Figures 9G,H. The results confirmed that scores of macrophages and T helper cells, cytolytic activity and type II IFN response were significant differences in the three clusters. From the above results, we can find that cluster 3 showed better survival outcomes, and we speculated the reason that cluster 3 could have stronger immunoreactivity.

### Validating expression of the seven genes in normal and osteosarcoma tissues

**FIGURE 10:**
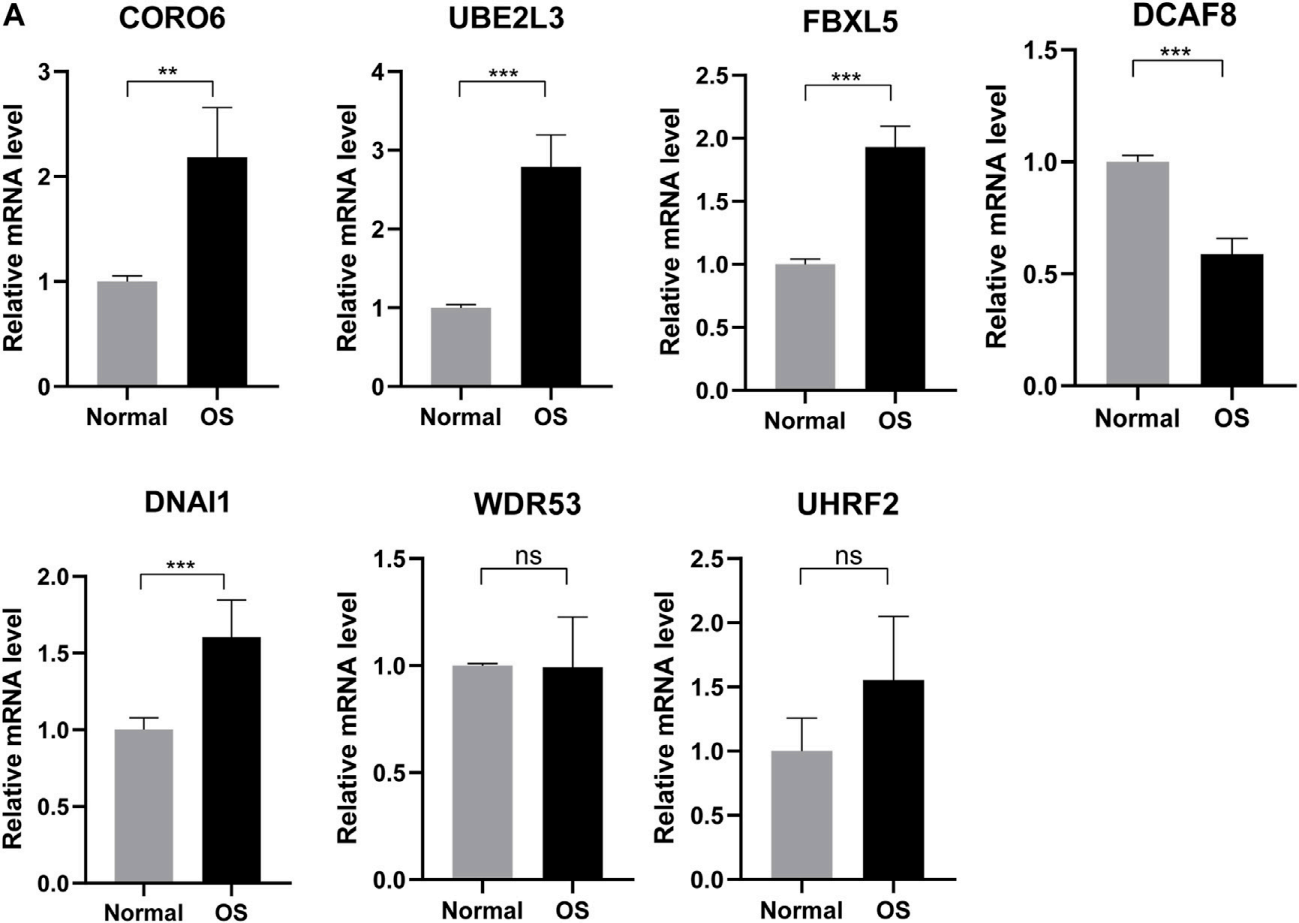
Validating expression of the seven genes in normal and osteosarcoma tissues. **(A)** The mRNA expression level of four ubiquitin-related genes (UBE2L3, CORO6, DCAF8, DNAI1, FBXL5, UHRF2, WDR53) in six normal and six osteosarcoma samples tissues. *p < 0.05, **p < 0.01*,* ***p < 0.001.

Based on better prediction efficiency, we further obtained differential expression of these seven genes in six normal and six osteosarcoma sample tissues through the qRT-PCR. The results showed that the mRNA expression level of four ubiquitin-related genes (CORO6, UBE2L3, FBXL5, DNAI1) was significantly increased in osteosarcoma tissues as compared with adjacent normal tissues. Moreover, the expression of DCAF8 was markedly decreased in osteosarcoma tissues than in adjacent normal tissues, and the expression of UHRF2 and WDR53 was not significant between osteosarcoma and normal tissues (Figure 10A). Based on the differential expression of these seven genes in clinical samples, it is necessary to explore the biological functions and mechanisms for OS treatment.

## Discussion

In recent years, a growing number of evidence has suggested that PTM was closely related to cell survival, cell cycle, differentiation, and innate and adaptive immunity for multiple cancer (Wu et al., 2019; Chen et al., 2020; Yang et al., 2020). As one of the most important PTM, ubiquitination has also attracted much attention in OS (Chen et al., 2014; Ying et al., 2016; Zhao et al., 2019). With the gradually increasing studies, regulating ubiquitination has been regarded as a novel therapeutic for OS (Jiang et al., 2020; Jiang et al., 2021; Mullard et al., 2021). Based on the above evidence, we further explored the significance of ubiquitin-related genes in OS prognosis through bioinformatic analysis.

In this study, we developed a ubiquitin-related gene signature including seven genes (UBE2L3, CORO6, DCAF8, DNAI1, FBXL5, UHRF2, WDR53) through univariate Cox regression, Lasso Cox regression, and multivariate Cox regression. Meanwhile, we confirmed that the gene signature had a good performance in predicting prognosis for OS patients. For the seven ubiquitin-related genes, there were a number of studies about their functions in human disease. For example, Tao et al. (2020) reported that overexpression of UBE2L3 could promote tumor progression through decreasing protein stability of GSK3β. Moreover, the small molecule PSSM0332 has been proved to inhibit inflammatory response by decreasing the DCAF8-medicated ubiquitination in the myocardial dysfunction model (Peng et al., 2020). Djakow et al. (2012) suggested that mutations DNAH5 and DNAI1 had an important significance in the diagnosis of primary ciliary dyskinesia. In addition, the research confirmed that FBXL5 could regulate HIF-1α activity by promoting the ubiquitination of CITED2 (Machado-Oliveira et al., 2015). Taken together, although there were a series of studies on seven ubiquitin-related genes, the reports associated with OS were poor. Hence, it will be necessary to explore their molecular biological functions in OS initiation and progression. In addition, many kinds of gene signature for OS were gradually increasing, such as immune-related gene signature (Xiao et al., 2020), hypoxia-associated prognostic signature (Fu et al., 2021), ferroptosis-related gene signature (Lei et al., 2021), and pyroptosis-related signature (Zhang et al., 2021). Among the above prognostic signature, our signature showed better predictive efficacy than others. Notably, there was still a need to verify the efficacy of the signature in the prospective study.

Currently, many studies suggested that ubiquitination had an important effect on the immune response. For example, increasing evidence demonstrated that targeting ubiquitination of programmed death 1 (PD-1)/programmed death-ligand 1 (PD-L1) could be a promising therapeutic approach for cancer immunotherapy (Hu et al., 2021). Hence, we explored the immune cells and immune-related functions between the high- and low-risk groups. Moreover, inflammation-promoting, T cell co-inhibition, and parainflammation exhibited a significant difference in the risk score model. Then, the results of GSEA showed some enrichment with immune. For instance, B cell receptor signaling pathway, complement activation, FC epsilon receptor signaling pathway, FC epsilon receptor-mediated stimulatory signaling pathway. In summary, the ubiquitin-related gene signature provided a good prediction for immune response.

All the time, doxorubicin, cisplatin, methotrexate, and paclitaxel are among the most chemotherapeutic agents for OS treatment (Patel et al., 1996; Bielack et al., 2015). However, a growing number of evidence indicated that resistance to standard chemotherapy is still a common problem for OS treatment. In our study, the drug sensitivity of the standard chemotherapy had no significance in the different risk models. Interestingly, IC50 of MG-132 showed a significant difference between high- and low-risk groups. Actually, emerging research have suggested that MG-132 played an important role in cancer chemotherapy (Pajonk et al., 2005; Li et al., 2007; Zhang et al., 2008). Moreover, Saini et al. (2021) have reported that MG-132 could be regarded as an effective therapeutic approach for OS treatment by inducing regulation of autophagy and protein homeostasis. MG-132 was also proved to modulate OS differentiation by regulating the expression of IRS-1 (Contaldo et al., 2014). Although some studies have verified that MG-132 has a pharmacological effect on OS treatment, it is necessary to explore the function of MG-132 in the clinical cohort.

In recent years, molecular subtypes have been proved to have instructive roles in preclinical and clinical therapy in many cancer types (Collisson et al., 2019). And in our study, we developed the three ubiquitination patterns through NMF algorithm. The results confirmed that cluster 3 showed better survival outcomes. Moreover, cluster 3 had the higher score in the stromal score, immune score, ESTIMATE score. Meanwhile, based on the relationship between ubiquitination and immune response, we compared the immune cell infiltration and immune-related functions in the three clusters. Overall, the above data indicated the probable conclusion that a better prognosis in cluster 3 was associated with stronger immunoreactivity.

Although we constructed a novel ubiquitin-related gene signature for predicting OS prognosis, there are still some limitations in this study. First, the ubiquitin-related gene signature only showed the relationship to OS prognosis, so it is necessary to explore their biological function for OS tumorigenesis. Second, due to the limitations of clinical factors, such as tumor grade and stage, we further demonstrated that the risk score model is an independent prognostic factor correlated with OS prognosis in large prospective clinical trials. Moreover, the clinical significance of OS subtypes should also be verified in the future.

In conclusion, we developed a novel ubiquitin-related gene signature which showed better predictive prognostic ability for OS and provided additional information on chemotherapy and immunotherapy. The OS molecular subtypes would also give a useful guide for individualized therapy.

## Data Availability

The datasets presented in this study can be found in online repositories. The names of the repository/repositories and accession number(s) can be found at: https://www.ncbi.nlm.nih.gov/geo/, GSE21257 https://www.ncbi.nlm.nih.gov/geo/, GSE39055.
