# Supplementary material for: A ubiquitin-related gene signature for predicting prognosis and constructing molecular subtypes in osteosarcoma
Source: Front Pharmacol. 2022 Aug 17;13:904448. doi: 10.3389/fphar.2022.904448 (PMC9428517; doi:10.3389/fphar.2022.904448)
Supplement: Supplementary file 2 [file Table2.docx]

Supplementary Table 2: All gene primers.

| Gene | Primer | Primer sequence (5′→3′) |
| --- | --- | --- |
| WDR53 | Forward | CGGGTGATGGGAGTTAAGTGTGAAC |
| WDR53 | Reverse | TGGGAGAAAGCAGACCTGGGATAC |
| UHRF2 | Forward | AGATGATGTTGAACCTGCTCCTTGG |
| UHRF2 | Reverse | CTGGGCTGCTTCTTTGACTGTCC |
| FBXL5 | Forward | CTTTGTCCTAACCTGGAGCATCTGG |
| FBXL5 | Reverse | AGCAACCAAGCCAAGACCAACTG |
| DNAI1 | Forward | TGGAGGCAGTAGGGGACACATTAG |
| DNAI1 | Reverse | GGTCTTCGAGGCATGGCAGTTC |
| DCAF8 | Forward | AACCGCACCAGCACAGAAAGTC |
| DCAF8 | Reverse | CCTCCTCTTCTTCCTCCTCTTCCTC |
| CORO6 | Forward | CCCGACTCCAGCATCGTCTA |
| CORO6 | Reverse | TAGCTTGTAGAACCGGGCGA |
| UBE2L3 | Forward | ACCCATTCAAACCACCGAAGATCAC |
| UBE2L3 | Reverse | GGCACTAATTACTGGCAGACAGACC |
