# Supplementary material for: A ubiquitin-related gene signature for predicting prognosis and constructing molecular subtypes in osteosarcoma
Source: Front Pharmacol. 2022 Aug 17;13:904448. doi: 10.3389/fphar.2022.904448 (PMC9428517; doi:10.3389/fphar.2022.904448)
Supplement: Supplementary file 3 [file Table3.docx]

Supplementary Table 3: Co-expression genes with ubiquitin related genes.

| **ubiquitination genes** | **co-expression genes** | **cor** | **pvalue** | **Regulation** |
| --- | --- | --- | --- | --- |
| CORO6 | CNB1 | 0.59 | 3.18E-09 | postive |
| CORO6 | PYGM | 0.52 | 3.24E-07 | postive |
| CORO6 | CAPN3 | 0.52 | 3.19E-07 | postive |
| CORO6 | DUPD1 | 0.51 | 5.54E-07 | postive |
| CORO6 | MBCA | 0.52 | 4.37E-07 | postive |
| DCAF8 | DUSP12 | 0.58 | 6.43E-09 | postive |
| DCAF8 | PPOX | 0.65 | 3.29E-11 | postive |
| DCAF8 | SDHC | 0.62 | 4.65E-10 | postive |
| DCAF8 | USP21 | 0.62 | 3.03E-10 | postive |
| DCAF8 | RRNAD1 | 0.60 | 2.05E-09 | postive |
| DCAF8 | USF1 | 0.65 | 1.64E-11 | postive |
| DCAF8 | NIT1 | 0.55 | 5.44E-08 | postive |
| DCAF8 | DEDD | 0.69 | 2.95E-13 | postive |
| DCAF8 | B4GALT3 | 0.57 | 1.60E-08 | postive |
| DCAF8 | PEX19 | 0.64 | 6.86E-11 | postive |
| DCAF8 | NCSTN | 0.54 | 1.49E-07 | postive |
| DCAF8 | CLK2 | 0.57 | 2.12E-08 | postive |
| DCAF8 | AL139011.1 | 0.50 | 1.27E-06 | postive |
| DCAF8 | ASH1L-AS1 | 0.54 | 9.75E-08 | postive |
| DCAF8 | AC103706.1 | 0.51 | 6.38E-07 | postive |
| DNAI1 | GRAMD1B | 0.54 | 1.44E-07 | postive |
| DNAI1 | MYOM2 | 0.51 | 9.52E-07 | postive |
| DNAI1 | CYFIP2 | 0.62 | 3.45E-10 | postive |
| DNAI1 | ROGDI | 0.51 | 8.64E-07 | postive |
| DNAI1 | FAP | -0.52 | 4.58E-07 | negative |
| DNAI1 | MEF2C | 0.52 | 3.47E-07 | postive |
| DNAI1 | HSPB11 | 0.52 | 3.05E-07 | postive |
| DNAI1 | ABLIM1 | 0.52 | 3.55E-07 | postive |
| DNAI1 | COCH | 0.53 | 1.93E-07 | postive |
| DNAI1 | SLC8A3 | 0.55 | 6.26E-08 | postive |
| DNAI1 | CDK6 | -0.52 | 5.13E-07 | negative |
| DNAI1 | FOLR1 | 0.56 | 2.43E-08 | postive |
| DNAI1 | DLX2 | 0.56 | 2.26E-08 | postive |
| DNAI1 | TMCC2 | 0.56 | 2.70E-08 | postive |
| DNAI1 | GRHL1 | 0.58 | 9.12E-09 | postive |
| DNAI1 | FAM189A2 | 0.60 | 1.54E-09 | postive |
| DNAI1 | CGREF1 | 0.53 | 1.60E-07 | postive |
| DNAI1 | ACP4 | 0.52 | 4.19E-07 | postive |
| DNAI1 | PANX3 | 0.52 | 5.27E-07 | postive |
| DNAI1 | COL6A3 | -0.51 | 8.23E-07 | negative |
| DNAI1 | EDIL3 | -0.50 | 1.21E-06 | negative |
| DNAI1 | WDR72 | 0.57 | 1.38E-08 | postive |
| DNAI1 | SEC11C | 0.55 | 4.44E-08 | postive |
| DNAI1 | TAC3 | 0.55 | 5.49E-08 | postive |
| DNAI1 | GNG4 | 0.51 | 7.19E-07 | postive |
| DNAI1 | ARMC4 | 0.55 | 8.23E-08 | postive |
| DNAI1 | ANO5 | 0.51 | 8.97E-07 | postive |
| DNAI1 | PHOSPHO1 | 0.51 | 8.10E-07 | postive |
| DNAI1 | PABPC5 | 0.50 | 1.08E-06 | postive |
| DNAI1 | DENND2C | 0.55 | 4.96E-08 | postive |
| DNAI1 | TAC4 | 0.57 | 1.39E-08 | postive |
| DNAI1 | ERBB4 | 0.52 | 5.02E-07 | postive |
| DNAI1 | UBE2E2 | -0.53 | 2.69E-07 | negative |
| DNAI1 | PRKX | 0.52 | 5.24E-07 | postive |
| DNAI1 | MYO6 | 0.51 | 9.61E-07 | postive |
| DNAI1 | C1orf137 | 0.57 | 1.45E-08 | postive |
| DNAI1 | COL11A2 | 0.53 | 1.93E-07 | postive |
| DNAI1 | FLJ45513 | 0.55 | 4.97E-08 | postive |
| DNAI1 | CASC10 | 0.51 | 6.05E-07 | postive |
| DNAI1 | AC092198.1 | 0.51 | 7.44E-07 | postive |
| DNAI1 | TNK2-AS1 | 0.50 | 1.19E-06 | postive |
| DNAI1 | AL451069.1 | 0.57 | 1.68E-08 | postive |
| DNAI1 | AC110015.1 | 0.57 | 1.64E-08 | postive |
| DNAI1 | ALG1L8P | 0.50 | 1.19E-06 | postive |
| DNAI1 | LINC01549 | 0.54 | 1.50E-07 | postive |
| DNAI1 | LINC01517 | 0.55 | 7.86E-08 | postive |
| DNAI1 | HMGA1P8 | 0.55 | 5.49E-08 | postive |
| DNAI1 | AL390026.1 | 0.52 | 5.15E-07 | postive |
| DNAI1 | CKMT1B | 0.52 | 3.47E-07 | postive |
| DNAI1 | AL512330.1 | 0.60 | 1.61E-09 | postive |
| DNAI1 | CORT | 0.59 | 2.43E-09 | postive |
| DNAI1 | AC009185.1 | 0.63 | 1.98E-10 | postive |
| DNAI1 | COX6B1P4 | 0.50 | 1.15E-06 | postive |
| DNAI1 | AC010609.1 | 0.62 | 2.19E-10 | postive |
| DNAI1 | AC027801.3 | 0.51 | 6.02E-07 | postive |
| DNAI1 | OR7E11P | 0.51 | 7.56E-07 | postive |
| DNAI1 | AP000997.1 | 0.63 | 1.88E-10 | postive |
| DNAI1 | AP003174.1 | 0.74 | 8.20E-16 | postive |
| DNAI1 | LINC02387 | 0.59 | 4.79E-09 | postive |
| DNAI1 | AC233723.1 | 0.54 | 1.14E-07 | postive |
| DNAI1 | PARD6G-AS1 | 0.57 | 1.07E-08 | postive |
| DNAI1 | S1PR2 | -0.50 | 1.13E-06 | negative |
| DNAI1 | AL355376.2 | 0.58 | 5.13E-09 | postive |
| DNAI1 | JMJD1C-AS1 | 0.55 | 6.22E-08 | postive |
| FBXL5 | CC2D2A | 0.58 | 1.00E-08 | postive |
| FBXL5 | RNF4 | 0.57 | 1.55E-08 | postive |
| FBXL5 | WDR1 | 0.57 | 1.46E-08 | postive |
| FBXL5 | ADD1 | 0.59 | 4.37E-09 | postive |
| FBXL5 | KLF16 | -0.51 | 5.92E-07 | negative |
| FBXL5 | TMEM128 | 0.50 | 1.23E-06 | postive |
| FBXL5 | RAB28 | 0.64 | 3.79E-11 | postive |
| FBXL5 | TAPT1 | 0.54 | 1.51E-07 | postive |
| FBXL5 | MRFAP1L1 | 0.52 | 4.39E-07 | postive |
| FBXL5 | HAUS3 | 0.53 | 1.59E-07 | postive |
| FBXL5 | FAM200B | 0.61 | 5.54E-10 | postive |
| FBXL5 | FAM111A-DT | 0.51 | 7.33E-07 | postive |
| UBE2L3 | UFD1 | 0.75 | 4.09E-16 | postive |
| UBE2L3 | CLTCL1 | 0.51 | 7.19E-07 | postive |
| UBE2L3 | DGCR2 | 0.57 | 1.08E-08 | postive |
| UBE2L3 | CDC45 | 0.61 | 6.88E-10 | postive |
| UBE2L3 | RANBP1 | 0.72 | 2.18E-14 | postive |
| UBE2L3 | KLHL22 | 0.55 | 5.77E-08 | postive |
| UBE2L3 | MED15 | 0.60 | 1.50E-09 | postive |
| UBE2L3 | SNAP29 | 0.67 | 2.20E-12 | postive |
| UBE2L3 | CRKL | 0.53 | 1.62E-07 | postive |
| UBE2L3 | SMARCB1 | 0.62 | 2.92E-10 | postive |
| UBE2L3 | BCL2L13 | 0.56 | 3.26E-08 | postive |
| UBE2L3 | PPIL2 | 0.72 | 1.45E-14 | postive |
| UBE2L3 | MAPK1 | 0.52 | 3.42E-07 | postive |
| UBE2L3 | PPM1F | 0.57 | 1.55E-08 | postive |
| UBE2L3 | ESS2 | 0.69 | 2.44E-13 | postive |
| UBE2L3 | SLC25A1 | 0.62 | 2.24E-10 | postive |
| UBE2L3 | HIRA | 0.75 | 3.11E-16 | postive |
| UBE2L3 | ANKRD54 | 0.57 | 1.34E-08 | postive |
| UBE2L3 | ASCC2 | 0.52 | 4.55E-07 | postive |
| UBE2L3 | SLC25A17 | 0.58 | 9.30E-09 | postive |
| UBE2L3 | CYB5B | 0.58 | 7.83E-09 | postive |
| UBE2L3 | DGCR6L | 0.61 | 5.20E-10 | postive |
| UBE2L3 | SDF2L1 | 0.59 | 2.72E-09 | postive |
| UBE2L3 | ATP6V1E1 | 0.52 | 4.99E-07 | postive |
| UBE2L3 | SLC2A11 | 0.50 | 1.18E-06 | postive |
| UBE2L3 | RAC1 | 0.52 | 5.22E-07 | postive |
| UBE2L3 | GUCD1 | 0.55 | 6.13E-08 | postive |
| UBE2L3 | ATP6V0D1 | 0.54 | 9.45E-08 | postive |
| UBE2L3 | HIC2 | 0.53 | 2.66E-07 | postive |
| UBE2L3 | METTL7B | 0.56 | 2.78E-08 | postive |
| UBE2L3 | TANGO2 | 0.65 | 1.73E-11 | postive |
| UBE2L3 | UQCR10 | 0.52 | 3.09E-07 | postive |
| UBE2L3 | THAP7 | 0.65 | 2.31E-11 | postive |
| UBE2L3 | TXNRD2 | 0.51 | 9.38E-07 | postive |
| UBE2L3 | ZNF74 | 0.51 | 6.12E-07 | postive |
| UBE2L3 | MRPL40 | 0.66 | 8.89E-12 | postive |
| UBE2L3 | DRG1 | 0.52 | 4.50E-07 | postive |
| UBE2L3 | GNB1L | 0.51 | 6.77E-07 | postive |
| UBE2L3 | PEX26 | 0.57 | 1.94E-08 | postive |
| UBE2L3 | AC097263.1 | 0.51 | 5.93E-07 | postive |
| UBE2L3 | C22orf39 | 0.52 | 3.80E-07 | postive |
| UBE2L3 | AC092718.4 | 0.53 | 1.73E-07 | postive |
| UBE2L3 | AC018371.2 | 0.55 | 4.88E-08 | postive |
| UBE2L3 | AL158801.5 | 0.52 | 3.72E-07 | postive |
| UHRF2 | CNTLN | 0.62 | 2.39E-10 | postive |
| UHRF2 | PICALM | 0.51 | 8.50E-07 | postive |
| UHRF2 | RBM7 | 0.50 | 1.13E-06 | postive |
| UHRF2 | RFX3 | 0.59 | 3.24E-09 | postive |
| UHRF2 | SMARCA2 | 0.55 | 4.71E-08 | postive |
| UHRF2 | PUM3 | 0.56 | 3.10E-08 | postive |
| UHRF2 | JAK2 | 0.61 | 9.80E-10 | postive |
| UHRF2 | ERMP1 | 0.55 | 5.39E-08 | postive |
| UHRF2 | CDC37L1 | 0.66 | 1.34E-11 | postive |
| UHRF2 | RIC1 | 0.69 | 3.31E-13 | postive |
| UHRF2 | KDM4C | 0.72 | 6.97E-15 | postive |
| UHRF2 | STRN | 0.52 | 5.23E-07 | postive |
| UHRF2 | RCL1 | 0.56 | 2.41E-08 | postive |
| UHRF2 | CAAP1 | 0.51 | 9.60E-07 | postive |
| UHRF2 | SMU1 | 0.52 | 5.32E-07 | postive |
| UHRF2 | STT3A | 0.55 | 4.38E-08 | postive |
| UHRF2 | RANBP6 | 0.71 | 3.89E-14 | postive |
| UHRF2 | DENND4C | 0.61 | 8.97E-10 | postive |
| UHRF2 | CREBZF | 0.53 | 2.13E-07 | postive |
| UHRF2 | AK3 | 0.66 | 7.45E-12 | postive |
| UHRF2 | HAUS6 | 0.67 | 3.91E-12 | postive |
| UHRF2 | CHEK1 | 0.51 | 6.60E-07 | postive |
| UHRF2 | RRAGA | 0.57 | 1.29E-08 | postive |
| UHRF2 | SNAPC3 | 0.63 | 1.09E-10 | postive |
| UHRF2 | PSIP1 | 0.59 | 3.84E-09 | postive |
| UHRF2 | PAFAH1B2 | 0.50 | 1.06E-06 | postive |
| UHRF2 | LIG4 | 0.53 | 2.11E-07 | postive |
| UHRF2 | ZDHHC21 | 0.57 | 1.38E-08 | postive |
| UHRF2 | GLDC | 0.55 | 6.98E-08 | postive |
| UHRF2 | TOPORS | 0.62 | 4.36E-10 | postive |
| UHRF2 | CHUK | 0.52 | 5.06E-07 | postive |
| UHRF2 | AL592293.2 | 0.51 | 7.59E-07 | postive |
| WDR53 | LSG1 | 0.56 | 2.61E-08 | postive |
| WDR53 | DZANK1 | 0.52 | 4.59E-07 | postive |
| WDR53 | AP3B2 | 0.51 | 7.82E-07 | postive |
| WDR53 | SCG3 | 0.54 | 1.02E-07 | postive |
| WDR53 | TBCCD1 | 0.55 | 5.24E-08 | postive |
| WDR53 | NCBP2 | 0.53 | 2.73E-07 | postive |
| WDR53 | SENP5 | 0.66 | 1.16E-11 | postive |
| WDR53 | CFAP58 | 0.51 | 7.23E-07 | postive |
| WDR53 | ZCCHC17 | 0.52 | 5.19E-07 | postive |
| WDR53 | ZNF576 | 0.51 | 5.47E-07 | postive |
| WDR53 | ACRV1 | 0.59 | 4.06E-09 | postive |
| WDR53 | USP44 | 0.64 | 6.35E-11 | postive |
| WDR53 | CCDC148 | 0.51 | 7.69E-07 | postive |
| WDR53 | SCN3A | 0.51 | 7.93E-07 | postive |
| WDR53 | PCYT1A | 0.54 | 9.45E-08 | postive |
| WDR53 | ERICH6 | 0.51 | 6.40E-07 | postive |
| WDR53 | SMIM12 | 0.50 | 1.09E-06 | postive |
| WDR53 | SHLD2P1 | 0.54 | 1.50E-07 | postive |
| WDR53 | NSG1 | 0.53 | 2.52E-07 | postive |
| WDR53 | SHLD1 | 0.51 | 7.43E-07 | postive |
| WDR53 | FBXO45 | 0.63 | 2.05E-10 | postive |
| WDR53 | MRPS23 | 0.62 | 2.49E-10 | postive |
| WDR53 | CCDC125 | 0.53 | 1.59E-07 | postive |
| WDR53 | RPL21P132 | 0.52 | 3.31E-07 | postive |
| WDR53 | SHLD2P3 | 0.51 | 7.40E-07 | postive |
| WDR53 | RNU6-200P | 0.50 | 1.10E-06 | postive |
| WDR53 | TCTEX1D2 | 0.59 | 4.97E-09 | postive |
| WDR53 | AC126120.1 | 0.53 | 2.47E-07 | postive |
| WDR53 | AL592293.1 | 0.51 | 5.47E-07 | postive |
| WDR53 | AC016769.2 | 0.51 | 5.92E-07 | postive |
| WDR53 | BCAS2P3 | 0.51 | 9.10E-07 | postive |
| WDR53 | LINC01063 | 0.55 | 7.60E-08 | postive |
| WDR53 | LINC02576 | 0.50 | 1.17E-06 | postive |
| WDR53 | AC018742.1 | 0.50 | 1.02E-06 | postive |
| WDR53 | HNRNPA1P42 | 0.51 | 6.07E-07 | postive |
| WDR53 | AC010343.2 | 0.52 | 3.94E-07 | postive |
| WDR53 | LINC02160 | 0.50 | 1.21E-06 | postive |
| WDR53 | AC068134.3 | 0.51 | 9.59E-07 | postive |
| WDR53 | AC106760.2 | 0.51 | 8.57E-07 | postive |
| WDR53 | AC104561.3 | 0.50 | 1.12E-06 | postive |
| WDR53 | AC004923.1 | 0.52 | 4.72E-07 | postive |
| WDR53 | DEFB130C | 0.51 | 6.02E-07 | postive |
| WDR53 | AC048337.1 | 0.54 | 1.19E-07 | postive |
| WDR53 | LINC02274 | 0.50 | 1.08E-06 | postive |
| WDR53 | AL359317.2 | 0.51 | 7.52E-07 | postive |
| WDR53 | DNAJC8P1 | 0.52 | 5.12E-07 | postive |
| WDR53 | AC107871.2 | 0.51 | 6.05E-07 | postive |
| WDR53 | AC027104.1 | 0.52 | 4.99E-07 | postive |
| WDR53 | AP005136.1 | 0.52 | 5.34E-07 | postive |
| WDR53 | MIR3122 | 0.52 | 4.11E-07 | postive |
| WDR53 | AP005136.2 | 0.51 | 8.06E-07 | postive |
| WDR53 | AC010201.2 | 0.50 | 1.14E-06 | postive |
| WDR53 | AC117490.2 | 0.56 | 2.43E-08 | postive |
| WDR53 | GHET1 | 0.59 | 2.55E-09 | postive |
