# Supplementary material for: A ubiquitin-related gene signature for predicting prognosis and constructing molecular subtypes in osteosarcoma
Source: Front Pharmacol. 2022 Aug 17;13:904448. doi: 10.3389/fphar.2022.904448 (PMC9428517; doi:10.3389/fphar.2022.904448)
Supplement: Supplementary file 4 [file DataSheet1.docx]

1. **Construction of a ubiquitin related genes signature**

library(survival)

pFilter=0.05

rt=read.table("expTime.txt",header=T,sep="\t",check.names=F,row.names=1)

rt$futime=rt$futime/365

outTab=data.frame()

sigGenes=c("futime","fustat")

for(gene in colnames(rt[,3:ncol(rt)])){

if(sd(rt[,gene])<0.01){

next}

a=rt[,gene]<=median(rt[,gene])

diff=survdiff(Surv(futime, fustat) ~a,data = rt)

pValue=1-pchisq(diff$chisq,df=1)

fit=survfit(Surv(futime, fustat) ~ a, data = rt)

cox=coxph(Surv(futime, fustat) ~ rt[,gene], data = rt)

coxSummary = summary(cox)

coxP=coxSummary$coefficients[,"Pr(>|z|)"]

if((pValue<pFilter) & (coxP<pFilter)){

sigGenes=c(sigGenes,gene)

outTab=rbind(outTab,

cbind(gene=gene,

KM=pValue,

B=coxSummary$coefficients[,"coef"],

SE=coxSummary$coefficients[,"se(coef)"],

HR=coxSummary$conf.int[,"exp(coef)"],

HR.95L=coxSummary$conf.int[,"lower .95"],

HR.95H=coxSummary$conf.int[,"upper .95"],

pvalue=coxP) )}}

write.table(outTab,file="uniCox.xls",sep="\t",row.names=F,quote=F)

uniSigExp=rt[,sigGenes]

uniSigExp=cbind(id=row.names(uniSigExp),uniSigExp)

write.table(uniSigExp,file="uniSigExp.txt",sep="\t",row.names=F,quote=F)

library("glmnet")

library("survival")

rt = read.table("uniSigExp.txt",header = T,sep = "\t",row.names = 1)

x <- as.matrix(rt[,c(3:ncol(rt))])

y <- data.matrix(Surv(rt$futime,rt$fustat))

fit <-glmnet(x, y, family="cox", maxit = 1000)

pdf(file="1.pdf",width=7,height=6)

plot(fit)

dev.off()

cvfit <-cv.glmnet(x, y, family="cox", maxit = 1000)

pdf(file="2.pdf",width=7,height=6)

plot(cvfit)

dev.off()

coef <- coef(fit, s = cvfit$lambda.min)

index <- which(coef != 0)

actCoef = coef[index]

lassoGene = row.names(coef)[index]

geneCoef = cbind(Gene = lassoGene,coef = actCoef)

write.table(geneCoef,file = "geneCoef.txt",sep = "\t",quote = F,row.names = F)

trainFinalGeneExp=rt[,lassoGene]

myFun=function(x){crossprod(as.numeric(x),actCoef)}

trainScore=apply(trainFinalGeneExp,1,myFun)

outCol=c("futime","fustat",lassoGene)

risk=as.vector(ifelse(trainScore>median(trainScore),"high","low"))

outTab=cbind(rt[,outCol],riskScore=as.vector(trainScore),risk)

write.table(cbind(id=rownames(outTab),outTab),file="Risk.txt",sep="\t",quote=F,row.names=F)

library(survival)

rt=read.table("Risk.txt",header=T,sep="\t",check.names=F,row.names=1)

multiCox=coxph(Surv(futime, fustat) ~ ., data = rt)

multiCox=step(multiCox,direction = "both")

multiCoxSum=summary(multiCox)

outTab=data.frame()

outTab=cbind(

coef=multiCoxSum$coefficients[,"coef"],

HR=multiCoxSum$conf.int[,"exp(coef)"])

outTab=cbind(id=row.names(outTab),outTab)

outTab=gsub("`","",outTab)

write.table(outTab,file="multiCox.txt",sep="\t",row.names=F,quote=F)

riskScore=predict(multiCox,type="risk",newdata=rt)

coxGene=rownames(multiCoxSum$coefficients)

coxGene=gsub("`","",coxGene)

outCol=c("futime","fustat",coxGene)

risk=as.vector(ifelse(riskScore>median(riskScore),"high","low"))

write.table(cbind(id=rownames(cbind(rt[,outCol],riskScore,risk)),cbind(rt[,outCol],riskScore,risk)),

file="risk.txt",

sep="\t",

quote=F,

row.names=F)

**2. Construction nomogram**

library(survival)

library(regplot)

library(rms)

riskFile="risk.all.txt"

cliFile="clinical.txt"

risk=read.table(riskFile, header=T, sep="\t", check.names=F, row.names=1)

cli=read.table(cliFile, header=T, sep="\t", check.names=F, row.names=1)

cli=cli[apply(cli,1,function(x)any(is.na(match('unknow',x)))),,drop=F]

cli$Age=as.numeric(cli$Age)

samSample=intersect(row.names(risk), row.names(cli))

risk1=risk[samSample,,drop=F]

cli=cli[samSample,,drop=F]

rt=cbind(risk1[,c("futime", "fustat", "risk")], cli)

res.cox=coxph(Surv(futime, fustat) ~ . , data = rt)

nom1=regplot(res.cox,

plots = c("density", "boxes"),

clickable=F,

title="",

points=TRUE,

droplines=TRUE,

observation=rt[15,],

rank="sd",

failtime = c(1,3,5),

prfail = F)

nomoRisk=predict(res.cox, data=rt, type="risk")

rt=cbind(risk1, Nomogram=nomoRisk)

outTab=rbind(ID=colnames(rt), rt)

write.table(outTab, file="nomoRisk.txt", sep="\t", col.names=F, quote=F)

pdf(file="calibration.pdf", width=5, height=5)

f <- cph(Surv(futime, fustat) ~ Nomogram, x=T, y=T, surv=T, data=rt, time.inc=1)

cal <- calibrate(f, cmethod="KM", method="boot", u=1, m=(nrow(rt)/3), B=1000)

plot(cal, xlim=c(0,1), ylim=c(0,1),

xlab="Nomogram-predicted OS (%)", ylab="Observed OS (%)", lwd=1.5, col="green", sub=F)

f <- cph(Surv(futime, fustat) ~ Nomogram, x=T, y=T, surv=T, data=rt, time.inc=3)

cal <- calibrate(f, cmethod="KM", method="boot", u=3, m=(nrow(rt)/3), B=1000)

plot(cal, xlim=c(0,1), ylim=c(0,1), xlab="", ylab="", lwd=1.5, col="blue", sub=F, add=T)

#5??У׼????

f <- cph(Surv(futime, fustat) ~ Nomogram, x=T, y=T, surv=T, data=rt, time.inc=5)

cal <- calibrate(f, cmethod="KM", method="boot", u=5, m=(nrow(rt)/3), B=1000)

plot(cal, xlim=c(0,1), ylim=c(0,1), xlab="", ylab="", lwd=1.5, col="red", sub=F, add=T)

legend('bottomright', c('1-year', '3-year', '5-year'),

col=c("green","blue","red"), lwd=1.5, bty = 'n')

dev.off()

**3. Immune cell infiltration and immune microenvironment**

#' @param X cell-specific gene expression

#' @param y mixed expression per sample

#' @export

CoreAlg <- function(X, y){

#try different values of nu

svn_itor <- 3

res <- function(i){

if(i==1){nus <- 0.25}

if(i==2){nus <- 0.5}

if(i==3){nus <- 0.75}

model<-svm(X,y,type="nu-regression",kernel="linear",nu=nus,scale=F)

model }

if(Sys.info()['sysname'] == 'Windows') out <- mclapply(1:svn_itor, res, mc.cores=1) else

out <- mclapply(1:svn_itor, res, mc.cores=svn_itor)

nusvm <- rep(0,svn_itor)

corrv <- rep(0,svn_itor)

#do cibersort

t <- 1

while(t <= svn_itor) {

weights = t(out[[t]]$coefs) %*% out[[t]]$SV

weights[which(weights<0)]<-0

w<-weights/sum(weights)

u <- sweep(X,MARGIN=2,w,'*')

k <- apply(u, 1, sum)

nusvm[t] <- sqrt((mean((k - y)^2)))

corrv[t] <- cor(k, y)

t <- t + 1}

#pick best model

rmses <- nusvm

mn <- which.min(rmses)

model <- out[[mn]]

#get and normalize coefficients

q <- t(model$coefs) %*% model$SV

q[which(q<0)]<-0

w <- (q/sum(q))

mix_rmse <- rmses[mn]

mix_r <- corrv[mn]

newList <- list("w" = w, "mix_rmse" = mix_rmse, "mix_r" = mix_r)}

doPerm <- function(perm, X, Y){

itor <- 1

Ylist <- as.list(data.matrix(Y))

dist <- matrix()

while(itor <= perm){

#print(itor)

#random mixture

yr <- as.numeric(Ylist[sample(length(Ylist),dim(X)[1])])

#standardize mixture

yr <- (yr - mean(yr)) / sd(yr)

#run CIBERSORT core algorithm

result <- CoreAlg(X, yr)

mix_r <- result$mix_r

#store correlation

if(itor == 1) {dist <- mix_r}

else {dist <- rbind(dist, mix_r)}

itor <- itor + 1}

newList <- list("dist" = dist)

}#' Main functions

CIBERSORT <- function(sig_matrix, mixture_file, perm=0, QN=TRUE){

library(e1071)

library(parallel)

library(preprocessCore)

#read in data

X <- read.table(sig_matrix,header=T,sep="\t",row.names=1,check.names=F)

Y <- read.table(mixture_file, header=T, sep="\t", row.names=1,check.names=F)

X <- data.matrix(X)

Y <- data.matrix(Y)

#order

X <- X[order(rownames(X)),]

Y <- Y[order(rownames(Y)),]

P <- perm #number of permutations

#anti-log if max < 50 in mixture file

if(max(Y) < 50) {Y <- 2^Y}

#quantile normalization of mixture file

if(QN == TRUE){

tmpc <- colnames(Y)

tmpr <- rownames(Y)

Y <- normalize.quantiles(Y)

colnames(Y) <- tmpc

rownames(Y) <- tmpr}

#intersect genes

Xgns <- row.names(X)

Ygns <- row.names(Y)

YintX <- Ygns %in% Xgns

Y <- Y[YintX,]

XintY <- Xgns %in% row.names(Y)

X <- X[XintY,]

#standardize sig matrix

X <- (X - mean(X)) / sd(as.vector(X))

#empirical null distribution of correlation coefficients

if(P > 0) {nulldist <- sort(doPerm(P, X, Y)$dist)}

#print(nulldist)

header <- c('Mixture',colnames(X),"P-value","Correlation","RMSE")

#print(header)

output <- matrix()

itor <- 1

mixtures <- dim(Y)[2]

pval <- 9999

#iterate through mixtures

while(itor <= mixtures){

y <- Y[,itor]

#standardize mixture

y <- (y - mean(y)) / sd(y)

#run SVR core algorithm

result <- CoreAlg(X, y)

#get results

w <- result$w

mix_r <- result$mix_r

mix_rmse <- result$mix_rmse

#calculate p-value

if(P > 0) {pval <- 1 - (which.min(abs(nulldist - mix_r)) / length(nulldist))}

#print output

out <- c(colnames(Y)[itor],w,pval,mix_r,mix_rmse)

if(itor == 1) {output <- out}

else {output <- rbind(output, out)} itor <- itor + 1 }

#save results

write.table(rbind(header,output), file="CIBERSORT-Results.txt", sep="\t", row.names=F, col.names=F, quote=F)

library("limma")

expFile="symbol.txt"

rt=read.table(expFile, header=T, sep="\t", check.names=F)

rt=as.matrix(rt)

rownames(rt)=rt[,1]

exp=rt[,2:ncol(rt)]

dimnames=list(rownames(exp),colnames(exp))

data=matrix(as.numeric(as.matrix(exp)),nrow=nrow(exp),dimnames=dimnames)

data=avereps(data)

data=data[rowMeans(data)>0,]

v <-voom(data, plot=F, save.plot=F)

out=v$E

out=rbind(ID=colnames(out), out)

write.table(out,file="uniq.symbol.txt",sep="\t",quote=F,col.names=F) #?????ļ?

results=CIBERSORT("ref.txt", "uniq.symbol.txt", perm=1000, QN=TRUE)

**3. nonnegative matrix factorization (NMF) clustering**

library(survival)

library(NMF)

setwd("C:\\biowolf\\NMF\\16.NMF")

rt=read.table("TCGA.expTime.txt", header=T, sep="\t", check.names=F, row.names=1)

sigGenes=c()

for(i in colnames(rt)[3:ncol(rt)]){

cox=coxph(Surv(futime, fustat) ~ rt[,i], data = rt)

coxSummary=summary(cox)

coxP=coxSummary$coefficients[,"Pr(>|z|)"]

if(coxP<0.01){ sigGenes=c(sigGenes,i) }

}

data=t(rt[,sigGenes])

res=nmf(data, rank=2:10, method="brunet", nrun=10, seed=123456)

pdf(file="cophenetic.pdf", width=8, height=7, onefile=F)

plot(res)

dev.off()

pdf(file="heatmap.all.pdf", width=15, height=15, onefile=F)

consensusmap(res,

annRow=NA,

annCol=NA,

#tracks=c("consensus:"),

main="Consensus matrix",

info=FALSE)

dev.off()

clusterNum=3

res=nmf(data, rank=clusterNum, method="brunet", nrun=10, seed=123456)

Cluster=predict(res)

Cluster=as.data.frame(Cluster)

Cluster$Cluster=paste0("C", Cluster$Cluster)

clusterOut=rbind(ID=colnames(Cluster), Cluster)

write.table(clusterOut, file="cluster.txt", sep="\t", quote=F, col.names=F)

pdf(file="heatmap.pdf", width=6, height=6, onefile=F)

consensusmap(res,

annRow=NA,

annCol=NA,

#tracks=c("consensus:"),

main="Consensus matrix",

info=FALSE)

dev.off()

**4. Prediction of drug sensitivity**

library(limma)

library(ggpubr)

library(pRRophetic)

library(ggplot2)

set.seed(12345)

expFile="symbol.txt"

riskFile="risk.all.txt"

allDrugs=c("A.443654", "A.770041", "ABT.263", "ABT.888", "AG.014699", "AICAR", "AKT.inhibitor.VIII", "AMG.706", "AP.24534", "AS601245", "ATRA", "AUY922", "Axitinib", "AZ628", "AZD.0530", "AZD2281", "AZD6244", "AZD6482", "AZD7762", "AZD8055", "BAY.61.3606", "Bexarotene", "BI.2536", "BIBW2992", "Bicalutamide", "BI.D1870", "BIRB.0796", "Bleomycin", "BMS.509744", "BMS.536924", "BMS.708163", "BMS.754807", "Bortezomib", "Bosutinib", "Bryostatin.1", "BX.795", "Camptothecin", "CCT007093", "CCT018159", "CEP.701", "CGP.082996", "CGP.60474", "CHIR.99021", "CI.1040", "Cisplatin", "CMK", "Cyclopamine", "Cytarabine", "Dasatinib", "DMOG", "Docetaxel", "Doxorubicin", "EHT.1864", "Elesclomol", "Embelin", "Epothilone.B", "Erlotinib", "Etoposide", "FH535", "FTI.277", "GDC.0449", "GDC0941", "Gefitinib", "Gemcitabine", "GNF.2", "GSK269962A", "GSK.650394", "GW.441756", "GW843682X", "Imatinib", "IPA.3", "JNJ.26854165", "JNK.9L", "JNK.Inhibitor.VIII", "JW.7.52.1", "KIN001.135", "KU.55933", "Lapatinib", "Lenalidomide", "LFM.A13", "Metformin", "Methotrexate", "MG.132", "Midostaurin", "Mitomycin.C", "MK.2206", "MS.275", "Nilotinib", "NSC.87877", "NU.7441", "Nutlin.3a", "NVP.BEZ235", "NVP.TAE684", "Obatoclax.Mesylate", "OSI.906", "PAC.1", "Paclitaxel", "Parthenolide", "Pazopanib", "PD.0325901", "PD.0332991", "PD.173074", "PF.02341066", "PF.4708671", "PF.562271", "PHA.665752", "PLX4720", "Pyrimethamine", "QS11", "Rapamycin", "RDEA119", "RO.3306", "Roscovitine", "Salubrinal", "SB.216763", "SB590885", "Shikonin", "SL.0101.1", "Sorafenib", "S.Trityl.L.cysteine", "Sunitinib", "Temsirolimus", "Thapsigargin", "Tipifarnib", "TW.37", "Vinblastine", "Vinorelbine", "Vorinostat", "VX680", "VX.702", "WH.4.023", "WO2009093972", "WZ.1.84", "X17.AAG", "X681640", "XMD8.85", "Z.LLNle.CHO", "ZM.447439")

rt = read.table(expFile, header=T, sep="\t", check.names=F)

rt=as.matrix(rt)

rownames(rt)=rt[,1]

exp=rt[,2:ncol(rt)]

dimnames=list(rownames(exp),colnames(exp))

data=matrix(as.numeric(as.matrix(exp)),nrow=nrow(exp),dimnames=dimnames)

data=avereps(data)

data=data[rowMeans(data)>0.5,]

group=sapply(strsplit(colnames(data),"\\-"), "[", 4)

group=sapply(strsplit(group,""), "[", 1)

group=gsub("2","1",group)

data=data[,group==0]

data=t(data)

rownames(data)=gsub("(.*?)\\-(.*?)\\-(.*?)\\-(.*)", "\\1\\-\\2\\-\\3", rownames(data))

data=avereps(data)

data=t(data)

riskRT=read.table(riskFile, header=T, sep="\t", check.names=F, row.names=1)

for(drug in allDrugs){

senstivity=pRRopheticPredict(data, drug, selection=1)

senstivity=senstivity[senstivity!="NaN"]

#senstivity[senstivity>quantile(senstivity,0.99)]=quantile(senstivity,0.99)

sameSample=intersect(row.names(riskRT), names(senstivity))

risk=riskRT[sameSample, "risk",drop=F]

senstivity=senstivity[sameSample]

rt=cbind(risk, senstivity)

rt$risk=factor(rt$risk, levels=c("low", "high"))

type=levels(factor(rt[,"risk"]))

comp=combn(type, 2)

my_comparisons=list()

for(i in 1:ncol(comp)){my_comparisons[[i]]<-comp[,i]}

test=wilcox.test(senstivity~risk, data=rt)

if(test$p.value<0.05){

boxplot=ggboxplot(rt, x="risk", y="senstivity", fill="risk",

xlab="Risk",

ylab=paste0(drug, " senstivity (IC50)"),

legend.title="Risk",

palette=c("#0066FF","#FF0000")

)+

stat_compare_means(comparisons=my_comparisons)

pdf(file=paste0("durgSenstivity.", drug, ".pdf"), width=5, height=4.5)

print(boxplot)

dev.off()

}

}
